# Age- and sex-matched comparison of diet quality in patients with heart failure to similarly aged healthy older adults

**DOI:** 10.1017/jns.2021.51

**Published:** 2021-08-18

**Authors:** JungHee Kang, Debra K. Moser, Martha J. Biddle, GYeon Oh, Terry A. Lennie

**Affiliations:** 1 College of Nursing, University of Kentucky, 2201 Regency Rd. Suite 403, Lexington, KY40503, USA; 2 Department of Pharmacy Practice and Science, College of Pharmacy, University of Kentucky, 789 South Limestone Street, Lexington, KY40536, USA; 3 College of Nursing, University of Kentucky, 751 Rose Street, Lexington, KY40536, USA

**Keywords:** Diet quality, Heart failure, Healthy eating index, Micronutrient deficiency, Older adults, HF, heart failure, HEI-2015, Healthy Eating Index-2015, BMI, body mass index, NDSR, Nutrition Data System for Research software

## Abstract

The aims of this study were to (1) compare diet quality between patients with heart failure (HF) and age- and sex-matched community-dwelling healthy older adults and (2) determine whether having HF was associated with a lower Healthy Eating Index-2015 (HEI-2015) score and risk of micronutrient deficiency. The HEI-2015 and macro- and micronutrient intakes of patients with HF were compared with healthy older adults (*N* 102; 55–92 years old; 53 % female). A paired *t*-test or Wilcoxon singed-rank test, McNemar's test, and conditional logistic regression were used to assess the association between diet quality and HF status. Median values for HEI-2015 and the number of micronutrient deficiency were used to dichotomise into groups in the conditional logistic regression. There was no significant between-group difference in the HEI-2015 total score (*P* 0⋅059), whereas the whole grain component was lower in patients with HF than in healthy older adults (3⋅1 ± 3⋅5 *v.* 4⋅5 ± 3⋅1, *P* 0⋅037; respectively). Total caloric intake was lower in patients with HF than in healthy older adults (1683 ± 595 *v.* 2104 ± 670 kcal; *P* < 0⋅001). Patients with HF had a higher average number of micronutrient deficiencies than healthy older adults (4[2, 6] *v.* 1[0, 4], respectively, *P* < 0⋅001). Patients with HF had four times higher odds of being in a high micronutrient deficiency group than healthy older adults, controlling for socio-demographics and body mass index (adjusted odds ratio [95 % confidence interval]: 4⋅04[1⋅06, 15⋅41]). Our findings demonstrate that diet quality measured by nutritional intake identifies patients with HF with lower caloric intake and higher micronutrient deficiencies compared with age- and sex-matched healthy older adults.

## Introduction

Heart failure (HF) is a growing epidemic that affected approximately 6⋅2 million individuals in the United States between 2013 and 2016^(1)^. The annual incidence of HF is 915 000, and the prevalence in adults is projected to increase by 46 % from 2012 to 2030 due to aging of the population^(2,3)^. Although major advances in medical management of HF have decreased symptom burden and improved outcomes, patients remain at risk for frequent rehospitalisations and death after optimisation of medical treatment^(4)^. Nutrition is among the non-pharmacologic factors that can complement medical treatment to improve HF outcomes^(5)^. However, most patients with HF need improvement in their diet quality^(6)^. We previously demonstrated that patients with HF who had higher number of dietary micronutrient deficiencies had double the risk of hospitalisation or death compared with those who had lower number of micronutrient deficiencies^(7)^. Most patients with HF are older, and older age is associated with consuming lower quality diet^(8)^. It is not known whether lower diet quality in patients with HF is primarily a function of older age or HF. To answer this question, we (1) compared diet quality (measured as Healthy Eating Index (HEI)-2015, dietary macronutrient and micronutrient intakes, and number of micronutrient deficiencies) between community-dwelling older patients with HF and age- and sex-matched healthy older adults, and (2) determined whether HF status was associated with lower HEI-2015 or higher risk of having dietary micronutrient deficiencies.

## Methods

### Study design

We used a cross-sectional design to compare data collected from an observational study of diet quality in community-dwelling patients with HF and healthy older adults. The parent study for the HF group was a longitudinal multicenter study examining nutrition, body mass index (BMI), and cardiac event-free survival in patients with HF. The parent study for the comparative group (healthy older adult) was a cross-sectional study designed to collect nutritional status, psychological, behavioural, and physical variables in a group of healthy community-dwelling older adults to compare patients with HF. These two groups were age- and sex-matched using a Statistical Analysis System Macro for one-to-one matching between patients with HF and healthy older adults and based on participants’ age (allowing ±1 year) and sex to reduce confounding^(9)^. This study was conducted according to the Declaration of Helsinki guidelines, and all procedures involving research study participants were approved by the University of Kentucky Institutional Review Boards. The study was thoroughly explained to all participants who then signed the written consent form.

### Sample

Participants with HF were recruited from midwestern and southeastern HF clinics and health older adults from a senior citizen centre. Patients with HF were eligible to participate in this study if they had (1) documented diagnosis of HF, (2) been stable on cardiac medications for at least 3 months, (3) no history of myocardial infarction or stroke within 3 months of enrolment and (4) no terminal or inflammatory comorbidity. Healthy older adults were eligible if they (1) were age ≥55 years, (2) had no obvious cognitive deficit as evidenced during conversation with the researcher and (3) had no history of myocardial infarction, coronary artery bypass surgery, angioplasty, HF, cancer, liver disease, gastrointestinal disease or end-stage renal disease. Participants were enrolled between May 2005 and March 2010.

### Measurements

#### Demographic variables and BMI

Age, sex, ethnicity, marital status, and financial status were collected via a self-reported questionnaire. For patients with HF, their status was confirmed from medical record review, as were the presence of comorbidities. Weight and height were obtained from all participants by research nurses in the clinical research centre. BMI was calculated as body weight in kilograms divided by height in metres squared.

#### Diet quality

Using 4-day food diaries (analysed using The Nutrition Data System for Research software [NDSR; Minneapolis, MN]), participants were asked to record all foods and liquids consumed for 1 weekend day and 3 weekdays. A trained research assistant visited participants’ homes to provide digital food scales with detailed oral and written instructions to weigh and record each food item consumed. Participants also received food models to estimate serving sizes in case they ate at a place (e.g., a restaurant) where they could not weigh their food. Participants were asked to provide a return demonstration of food measurement and recording in their food diary to ensure the accuracy of 4-day food diaries. On the first day of the food diary collection, a research assistant called participants to review the procedure and answer any questions they had. Participants’ 4-day food diaries were reviewed with participant the morning after completion by a registered dietitian for the verification of serving sizes, food preparation methods, and to collect any missing data. The following two indicators of diet quality were derived from the 4-day food diaries: (1) the HEI-2015 and (2) macro- and micronutrient intakes, and number of dietary micronutrient deficiencies.

##### Healthy Eating Index-2015

The HEI assess whether foods consumed align with the Dietary Guidelines for Americans (DGA)^(10)^. The HEI-2015^(11)^ version of the HEI reflects the 2015–2020 DGA. The HEI is composed of scores for thirteen dietary components. Nine ‘adequacy’ components are those recommended to consume by the United States Department of Agriculture: total fruits (five points), whole fruits (five points), total vegetables (five points), greens and beans (five points), whole grains (ten points), dairy (ten points), total protein foods (five points), seafood and plant proteins (five points), and fatty acid ratio (ten points). Four ‘moderation’ components are those recommended for consumption in limited amounts are scored in reverse with lower consumption associated with higher scores: refined grains (ten points), sodium (ten points), saturated fats (ten points), and added sugars (ten points). The total HEI-2015 score is the sum of thirteen component scores and can range from 0 to 100. Higher scores indicate closer reflects closer conformance with dietary guidance, and thus, better diet quality.

The HEI-2015 is calculated based on a 1000 kcal/d formula to characterise diet quality while controlling for diet quantity and addresses the consumption of energy-dense and nutrient-poor food and ingredients. The NDSR software, developed by the Nutrition Coordination Center (NCC), was used to analyse 4-day food diary data and create variables needed to calculate scores for each component of the HEI-2015^(12)^. Briefly, average daily intake of each food subgroup was calculated from the NDSR output data^(13)^. Total servings were adjusted (i.e. cups, ounces, grams, or % energy) according to the Nutrition Coordinating Center Food Serving Count System. The adjusted servings were divided by energy intake to generate servings of HEI-2015 components per 1000 kcal. The final scores of each component were generated linearly based on the standards for maximum and minimum scores^(14)^. Validity and reliability of the HEI-2015 were demonstrated using 24 h recalls from the National Health and Nutrition Examination Survey 2011–2012 conducted by the Centers for Disease Control and Prevention and National Center for Health Statistics^(15)^. Reedy *et al.* reported that HEI-2015 scores were at or very near the maximum levels for most sets of exemplary menus.

##### Macro- and micronutrient diet quality

###### Macronutrient distribution

Intake of carbohydrate, protein, and fat by grams and as percentage of total kcal were obtained from the NDSR output^(16)^.

###### Micronutrient intake

Four-day averaged dietary intake of micronutrients was obtained from the NDSR output. The number of eleven vitamins (thiamin, riboflavin, niacin, pantothenic acid, vitamin B_6_, folate, vitamin B_12,_ vitamin C, vitamin D, vitamin E and vitamin K) and six minerals (calcium, magnesium, selenium, iron, zinc and phosphorus) defined as deficient in the diet were compared between healthy older adults and patients with HF^(7)^. Micronutrient intake was also calculated based on 1000 kcal/d for the qualitative comparison. Dietary deficiency of thiamin, riboflavin, niacin, vitamin B_6_, folate, magnesium and phosphorus was determined using the probability formula recommended by the National Academy of Medicine^(17–19)^. A probability score of less than −1 indicated that, based on the 4-day averaged intake, there was a high probability the individual's diet was habitually deficient in that nutrient. Because probability formulas could not be used due to insufficient population data, dietary deficiency of calcium, iron, selenium, zinc, vitamin B_12_, vitamin C, vitamin D and vitamin E was determined by dividing average intake by estimated average requirement. Deficiency was defined as a ratio less than 1⋅0. Pantothenic acid and vitamin K deficiency were defined as average intake <50 % of the adequate intake value for each nutrient, because estimated average requirement values were not available for these two micronutrients. The number of micronutrients defined as deficient was totalled to determine nutrient diet quality.

### Data analysis

We used SAS software for data analyses (version 9.4). Age- and sex-matched data between healthy older adults and patients with HF were horizontally combined to compare sample characteristics, HEI-2015 scores, average intake of macronutrients, average intake of micronutrients and the proportions of micronutrient deficiencies between healthy older adults and patients with HF. Because age- and sex-matched groups are no longer considered independent, a McNemar's test or an Exact test for symmetry was used to compare categorical variables between the two groups. The Shapiro–Wilk test was used to test the normality of continuous variables. A paired sample *t*-test was used for the variables when the mean difference between healthy older adults and patients with HF was normally distributed, while the Wilcoxon signed-rank test was applied when the mean difference between healthy older adults and patients with HF did not meet the normal distribution assumption. For the logistic regression, age- and sex-matched data between healthy older adults and patients with HF were vertically combined. Conditional logistic regression was used to examine the relationship between HF *v*. healthy status and diet quality defined by HEI-2015 scores and dietary micronutrient deficiencies, respectively. As a response variable, HEI-2015 scores were categorised into two groups at the median value: higher quality diet group (higher HEI-2015 score) and lower quality diet group (lower HEI-2015 score). To use the number of dietary micronutrient deficiency as a response variable, we created a binary variable using a cut-off point 2 as the high micronutrient deficiency group. Participants with zero to one micronutrient deficiency were classified as the low deficiency group. Marital status, BMI, financial status, and living arrangements were used as covariates in adjusted conditional regression models. Statistical significance was set at *P* < 0⋅05; all tests were two-sided.

## Results

### Sample characteristics

The mean age of participants was 69⋅9 ± 8⋅6 (range: 55–92) years old and 53 % were female. Comparisons of participant characteristics between the two groups are shown in Table 1. McNemar's test and an Exact test for symmetry indicated that there was a lower proportion of married/cohabitating and a higher proportion of “not enough to make ends meet” in patients with HF compared with age- and sex-matched healthy older adults (*P* 0⋅025 and *P* < 0⋅001, respectively). Patients with HF had a higher average BMI than healthy older adults (29⋅2 ± 6⋅8 *v.* 26⋅4 ± 4⋅6 kg/m^2^; *P* 0⋅018). There was a higher proportion of hypertension and diabetes mellitus in patients with HF compared with healthy older adults (*P* < 0⋅001).

**Table 1.** Participant characteristics (*N* 102)

Characteristics		Mean [sd] or *n* (%)		*P*-value
	Total (*N* 102)^†^	Healthy adults (*n* 51)	Heart failure (*n* 51)	
Sex (female)	54 (53)	27 (53)	27 (53)
Age (years)^‡^	69⋅9 [8⋅6]	69⋅8 [8⋅6]	69⋅9 [8⋅6]	0⋅806
Ethnicity (Caucasian)^§^	90 (88)	46 (90)	44 (86)	0⋅564
Marital status (married/cohabitating)^§^	61 (61)	35 (71)	25 (51)	0⋅025
Financial status^§^				
Comfortable	56 (54⋅9)	38 (74⋅5)	18 (35⋅3)	<0⋅001
Enough to make ends meet	35 (34⋅3)	12 (23⋅5)	23 (45⋅1)	
Not enough to make ends meet	11 (10⋅8)	1 (2⋅0)	10 (19⋅6)	
BMI (kg/m^2^)^‡^	27⋅8 [6⋅0]	26⋅4 [4⋅6]	29⋅2 [6⋅8]	0⋅018
Hypertension (yes)^§^	54 (54⋅5)	14 (28)	40 (78)	<0⋅001
Diabetes mellitus (yes)^§^	27 (27)	5 (10)	22 (43)	<0⋅001

sd, standard deviation.

† Sample size may be less for some variables due to missing data.

‡ Paired *t*-test was used.

§ The McNemar's test or an Exact test for symmetry was used.

### Diet quality by HEI-2015 scores

As shown in Table 2, there was no statistically significant difference in total HEI-2015 scores between patients with HF and healthy older adults (54⋅9 ± 11⋅7 *v.* 59⋅79 ± 12⋅6, respectively, *P* 0⋅059). There were no differences in adequacy components between patients with HF and healthy older adults, except the intake of whole grains was significantly lower in patients with HF than in healthy older adults (3⋅1 ± 3⋅5 *v*. 4⋅5 ± 3⋅1, respectively, *P* 0⋅037). None of the moderation components or total HEI-2015 scores was different between patients with HF and healthy older adults.

**Table 2.** HEI-2015 scores between age- and sex-matched healthy older adults and patients with heart failure (*N* 102)

HEI-2015 components	Healthy adults (*n* 51)		Heart failure (*n* 51)		*P*-value
	Mean or (median)	sd or [Q1, Q3]	Mean or (median)	sd or [Q1, Q3]	
Total HEI-2015^†^	59⋅79	12⋅6	54⋅9	11⋅7	0⋅059
Total fruits (cups)^†^	2⋅7	1⋅7	2⋅3	1⋅9	0⋅240
Whole fruits (cups)^†^	3⋅2	1⋅6	2⋅6	2⋅0	0⋅095
Total vegetables (cups)^†^	3⋅8	1⋅1	3⋅5	1⋅5	0⋅264
Greens and beans (cups)^†^	3⋅4	1⋅8	2⋅8	2⋅0	0⋅148
Whole grains (oz)^†^	4⋅5	3⋅1	3⋅1	3⋅5	0⋅037
Dairy (cups)^†^	5⋅8	2⋅3	5⋅6	3⋅4	0⋅715
Total protein (oz)^‡^	(5⋅0)	[5⋅0, 5⋅0]	(5⋅0)	[4⋅8, 5⋅0]	0⋅814
Seafood and plant proteins (oz)^‡^	(5⋅0)	[2⋅4, 5⋅0]	(4⋅6)	[1⋅8, 5⋅0]	0⋅603
Fatty acid ratio	4⋅7	3⋅3	4⋅7	3⋅1	0⋅913
Refined grains (oz)^†^	7⋅4	3⋅1	6⋅9	2⋅7	0⋅456
Sodium (g)^†^	4⋅0	2⋅9	3⋅4	3⋅1	0⋅364
Added sugars (% energy)^†^	6⋅6	2⋅8	6⋅9	3⋅2	0⋅548
Saturated fat (% energy)^†^	5⋅3	3⋅4	4⋅8	3⋅0	0⋅393

HEI, Healthy Eating Index-2015; sd, standard deviation; Q1, the first quartile; Q3, the third quartile; oz, ounce.

† Determined using paired *t*-tests.

‡ Determined using Wilcoxon signed-rank tests.

### Macronutrient composition of diet

Patients with HF had a lower total caloric intake (1683 ± 595 *v.* 2104 ± 670 kcal; *P* < 0⋅001) than healthy older adults (Table 3). Patients with HF also had lower carbohydrate (195 ± 71 *v.* 256 ± 91 g, *P* < 0⋅001), protein (68 ± 23 *v.* 81 ± 20 g, *P* 0⋅035) and fat intakes (71 ± 34 *v.* 86 ± 37 g, *P* 0⋅038) compared with healthy older adults. The caloric intakes of animal and vegetable protein subcomponents and unsaturated fat subcomponents were also lower in patients with HF. However, there were no significant differences in percent of food energy from carbohydrate, protein and fat between patients with HF and healthy older adults.

**Table 3.** Comparison of macronutrients between age- and sex-matched healthy older adults and patients with heart failure (*N* 102)

	Healthy Adults (n = 51)		Heart Failure (n = 51)		*P*-value
Variables	Mean or Median	sd or (Q1, Q3)	Mean or Median	sd or (Q1, Q3)	
*Absolute values of macronutrients*					
Total energy intake (kcal)^†^	2,104	670	1,683	595	<⋅001
Carbohydrate (g)^†^	255⋅7	90⋅5	195⋅3	71⋅1	<⋅001
Protein (g)^†^	80⋅6	19⋅84	68⋅12	23⋅04	⋅0035
Animal protein (g)^‡^	50	(43, 62)	45	(33, 61)	<⋅001
Vegetable protein (g)^‡^	25	(20, 32)	21	(15, 24)	<⋅001
Fat (g)^†^	86⋅2	37	71⋅5	33⋅9	0⋅038
Saturated fat (g)^‡^	25	(20, 33)	21	(15, 29)	<⋅001
Monosaturated fat (g)^‡^	30	(23, 41)	23	(18, 35)	<⋅001
Polysaturated fat (g)^‡^	15	(11, 21)	12	(10, 18)	<⋅001
*Percent (%) of food energy*						
Carbohydrate^†^	49⋅3	7⋅4	47⋅1	7⋅5	0⋅120
Protein^†^	16⋅2	3⋅6	16⋅8	3⋅4	0⋅350
Animal protein (g)^‡^	10⋅6	3⋅4	11⋅4	3⋅5	0⋅223
Vegetable protein (g)^‡^	5⋅3	(4⋅1, 6⋅3)	4⋅9	(4⋅1, 5⋅8)	0⋅135
Fat^†^	35⋅5	6⋅4	36⋅7	6⋅8	0⋅330
Saturated fat^‡^	11⋅1	(9⋅5, 14⋅6)	12⋅3	(10⋅4,13⋅9)	0⋅381
Monosaturated fat^†^	13⋅7	3⋅2	14⋅1	3⋅5	0⋅454
Polysaturated fat^†^	7⋅3	2⋅2	7⋅4	2⋅1	0⋅760

sd, standard deviation; Q1, the first quartile; Q3, the third quartile.

† Determined using paired *t*-tests.

‡ Determined using Wilcoxon signed-rank tests.

### Micronutrient composition

Average daily intakes of calcium, magnesium, vitamin D, vitamin C, vitamin K, folate, pantothenic acid, vitamin B_6_, selenium, phosphorus, niacin, thiamin and riboflavin were lower in patients with HF compared with healthy older adults (Table 4). The proportion of participants with diets deficient in calcium (73 *v.* 49 %), magnesium (73 *v.* 28 %), vitamin D (73 *v*. 45 %), vitamin C (39 *v*. 18 %) and folate (35 *v*. 16 %) was higher in patients with HF compared with healthy older adults (*P* 0⋅019, *P* < 0⋅001, *P* 0⋅011, *P* 0⋅022 and *P* 0⋅025, respectively). Patients with HF had a higher average number of micronutrient deficiencies compared with healthy older adults (4 [2, 6] *v*. 1 [0, 4], respectively, *P* < 0⋅001). When we adjusted micronutrient intake per 1000 kcal/d, magnesium (174 [159, 192] *v*. 152 [140, 165], *P* 0⋅037) and vitamin C (74⋅8 [56⋅6, 98⋅7] *v.* 48⋅6 [35⋅4, 66⋅6], *P* 0⋅049) were still significantly lower in patients with HF compared with healthy older adults (Table 5).

**Table 4.** Summary of the micronutrients, including geometric means and their corresponding 95 % confidence intervals and percentage of the deficiency between age- and sex-matched healthy adults and patients with heart failure groups (*N* 102)

Variable	Geomean (95 % CI)		*P*-value	% Deficient^†^		*P*-value^‡^
	Healthy (*n* 51)	HF (*n* 51)		Healthy	HF	
Calcium^§^	1064 (943, 1 201)	730 (619, 861)	<0⋅001^‖^	49⋅0	72⋅6	0⋅019
Magnesium^¶^	351 (317, 390)	242 (214, 274)	<0⋅001^‖^	27⋅5	72⋅6	<0⋅001
Vitamin D^§^	8⋅6 (6⋅6, 11⋅2)	5 (3⋅9, 6⋅6)	0⋅011^‖^	45⋅1	72⋅6	0⋅011
Vitamin E^§^	19⋅1 (13⋅2, 27⋅7)	13 (8⋅6, 19⋅4)	0⋅203^‖^	43⋅1	58⋅8	0⋅131
Zinc^§^	13⋅9 (11⋅8, 16⋅3)	11⋅6 (9⋅5, 14⋅2)	0⋅164^‖^	33⋅3	51⋅0	0⋅061
Vitamin C^§^	150⋅6 (113⋅6, 199⋅6)	77⋅2 (54⋅6, 109⋅2)	0⋅004^‖^	17⋅7	39⋅2	0⋅022
Vitamin K**	105⋅1 (89⋅2, 123⋅9)	75⋅8 (61⋅2, 93⋅9)	0⋅016^‖^	35⋅3	51⋅0	0⋅103
Folate^¶^	680 (477, 968)	416 (351, 493)	0⋅001^††^	15⋅7	35⋅3	0⋅025
Vitamin B_12_^§^	12⋅2 (6⋅4, 23⋅2)	7⋅1 (4⋅1, 12⋅3)	0⋅073^††^	3⋅9	16⋅0	0⋅058
Pantothenic acid**	9⋅5 (7⋅7, 11⋅8)	6⋅2 (5⋅2, 7⋅4)	0⋅002^‖^	17⋅7	25⋅5	0⋅285
Vitamin B_6_^¶^	3⋅2 (2⋅5, 4)	2 (1⋅6, 2⋅3)	<0⋅001^‖^	2	7⋅8	0⋅375
Selenium^§^	157⋅8 (100⋅5, 247⋅8)	91⋅5 (79⋅9, 104⋅9)	0⋅002^††^	0	7⋅8	−
Iron^§^	17⋅2 (15, 19⋅6)	15⋅2 (12⋅4, 18⋅5)	0⋅232^‖^	0	3⋅9	−
Phosphorus^¶^	1324 (1228, 1427)	1043 (937, 1162)	<0⋅001^‖^	0	5⋅9	−
Niacin^¶^	36⋅1 (28⋅7, 45⋅4)	23⋅4 (19⋅4, 28⋅2)	0⋅001^††^	0	5⋅9	−
Thiamin^¶^	2⋅5 (2, 3)	1⋅7 (1⋅5, 1⋅9)	<0⋅001^††^	0	2⋅0	−
Riboflavin^¶^	3⋅2 (2⋅7, 3⋅9)	2⋅3 (2, 2⋅6)	0⋅005^††^	0	0	−

CI, confidence interval; HF, heart failure.

Dietary micronutrient intakes were calculated as 4-day average.

Geomean was calculated as the result of back transformation of the mean of the logarithms of dietary micronutrient intakes.

For calcium, magnesium, vitamin E, zinc, vitamin C, pantothenic acid, vitamin B6, iron, phosphorus, niacin, thiamin and riboflavin, milligram was used as a unit of measurement, while microgram was used as a unit of measurement for vitamin D, vitamin K, folate, vitamin B12 and selenium.

† The McNemar's test was used when comparing micronutrient deficiency between patients with HF and healthy older adults. An Exact test was used when the number of discordant pairs was less than 10.

‡ The McNemar's test was used.

§ Micronutrient deficiency status based on the estimated average requirement.

‖ Determined using paired *t*-tests for the log-transformed micronutrients.

¶ Micronutrient deficiency status based on probability formula.

** Micronutrient deficiency status based on <50 % adequate intake value.

†† Determined using Wilcoxon signed-rank tests for the log-transformed micronutrients.

**Table 5.** Summary of the micronutrients, including geometric means and their corresponding 95 % confidence intervals after adjusting for 1000 kcal/d between age- and sex-matched healthy adults and patients with heart failure groups (*N* 102)

Variable	Geomean (95 % CI)		*P*-value
	Healthy (*n* 51)	HF (*n* 51)	
Calcium	528 (465, 600)	459 (400, 526)	0⋅162^†^
Magnesium	174 (159, 192)	152 (140, 165)	0⋅037^†^
Vitamin D	4⋅3 (3⋅3, 5⋅5)	3⋅2 (2⋅5, 4⋅1)	0⋅142^†^
Vitamin E	9⋅5 (6⋅7, 13⋅5)	8⋅2 (5⋅5, 12)	0⋅600^†^
Zinc	6⋅9 (5⋅8, 8⋅2)	7⋅3 (6⋅2, 8⋅6)	0⋅634^†^
Vitamin C	74⋅8 (56⋅6, 98⋅7)	48⋅6 (35⋅4, 66⋅6)	0⋅049^†^
Vitamin K	52⋅2 (43⋅7, 62⋅4)	47⋅7 (38⋅4, 59⋅1)	0⋅495^†^
Folate	337 (235, 484)	262 (225, 303)	0⋅219^‡^
Vitamin B_12_	6⋅1 (3⋅2, 11⋅6)	4⋅5 (2⋅7, 7⋅4)	0⋅566^‡^
Pantothenic acid	4⋅8 (3⋅8, 5⋅9)	3⋅9 (3⋅3, 4⋅6)	0⋅140^†^
Vitamin B_6_	1⋅6 (1⋅3, 2)	1⋅2 (1⋅1, 1⋅4)	0⋅130^‡^
Selenium	78⋅4 (50⋅6, 121⋅3)	57⋅5 (52⋅7, 62⋅9)	0⋅541^‡^
Iron	8⋅5 (7⋅5, 9⋅7)	9⋅6 (8, 11⋅5)	0⋅291^†^
Phosphorus	657 (618, 699)	656 (615, 700)	0⋅488^‡^
Niacin	17⋅9 (14⋅3, 22⋅5)	14⋅7 (12⋅5, 17⋅3)	0⋅241^‡^
Thiamin	1⋅2 (1, 1⋅5)	1⋅1 (1, 1⋅2)	0⋅177^‡^
Riboflavin	1⋅6 (1⋅3, 2)	1⋅4 (1⋅3, 1⋅6)	0⋅573^‡^

CI, confidence interval; HF, heart failure.

Dietary micronutrient intakes were calculated as 4-day average.

Geomean was calculated as the result of back transformation of the mean of the logarithms of dietary micronutrient intakes per 1000 kcal/d.

For calcium, magnesium, vitamin E, zinc, vitamin C, pantothenic acid, vitamin B6, iron, phosphorus, niacin, thiamin and riboflavin, milligram was used as a unit of measurement, while microgram was used as a unit of measurement for vitamin D, vitamin K, folate, vitamin B12 and selenium.

† Determined using paired *t*-tests for the energy-adjusted log-transformed micronutrients.

‡ Determined using Wilcoxon signed-rank tests for the energy-adjusted log-transformed micronutrients.

### Association of HF status and diet quality

In the conditional logistic regression model, we regressed diet quality on HF status using total HEI-2015 scores (cut-off point: median value 57⋅6) and the status of micronutrient (≤1 *v.* >1) deficiencies while controlling for marital status, BMI, financial status and living arrangements in age- and sex-matched community-dwelling older adults (Table 6). There was no significant association between HF status and HEI-2015 in either the unadjusted or adjusted model (*P* 0⋅079 or *P* 0⋅751, respectively). However, the model indicated a statistically significant association between HF status and dietary micronutrient deficiency in both unadjusted (bivariate) and adjusted (controlling for marital status, BMI, financial status and living arrangements) models (*P* 0⋅004 and *P* 0⋅041, respectively). In the unadjusted model, the estimated odds of patients with HF being in the high micronutrient deficiency group was 3⋅4 times higher than similarly aged healthy older adults (95 % confidence interval [1⋅48, 7⋅96]). While holding covariates constant, the estimated odds of patients with HF being in the high micronutrient deficiency group was four times higher than similarly aged healthy older adults in the adjusted model (95 % confidence interval [1⋅06, 15⋅41]).

**Table 6.** Association between heart failure status and diet quality using HEI-2015 and micronutrient deficiency in age- and sex-matched older adults (*N* 99)

	HEI-2015		Micronutrient deficiency	
	OR (95 % CI)	*P*-value	OR (95 % CI)	*P*-value
Heart failure status				
Unadjusted^†^	2⋅13 (0⋅92, 4⋅92)	0⋅079	3⋅43 (1⋅48, 7⋅96)	0⋅004
Adjusted^‡^	0⋅79 (0⋅19, 3⋅38)	0⋅751	4⋅04 (1⋅06, 15⋅41)	0⋅041

OR, odds ratio; CI, confidence interval.

† Unadjusted model.

‡ Adjusted model. Conditional logistic regression model was used for the analysis. The HEI-2015 scores were divided into two groups at the median value. The number of dietary micronutrient deficiency was divided into two groups using a cut-off point of >1. Marital status (married/cohabitating, single/divorced/separated/widowed), BMI (kg/m^2^; continuous), financial status (comfortable, not comfortable) and living arrangements (live alone, live with someone) were controlled in the adjusted model.

## Discussion

The HEI has become a common method of comparing diet quality across multiple populations^(20–23)^. The HEI-2015 was similar between the patients with HF and healthy older adults, except whole grains. In contrast, when we compared macro- and micronutrient intakes, we found lower macronutrient and micronutrient intakes in patients with HF compared with age- and sex-matched healthy older adults. These significant differences in nutrient intakes diminished when we compared macronutrient intake as percent food energy and micronutrient intake based on 1000 kcal/d except in the case of magnesium and vitamin C. In addition, we found a higher number of micronutrient deficiencies in patients with HF compared with the healthy older adults. Two conclusions are suggested from these data. First, diet quality is an important factor to consider in patients with HF. Second, HF, rather than age, appears to play an important role in diet quality, defined by micronutrient deficiency in patients with HF.

HF often co-exists with lower diet quality, which is associated with increased rehospitalisation and mortality^(7,24,25)^. Wu *et al.* found an association of vitamin C deficiency with health-related quality of life and cardiac event-free survival^(24)^. Insufficient nutrition with poor health outcomes can also occur in older adults^(26,27)^. The results of our study indicated that older patients with HF were at higher risk of having micronutrient deficiencies than sex- and age-matched healthy older adults. The potential reasons for lower diet quality in those with HF include polypharmacy, HF symptoms, the impact of depressive symptoms, and dietary restrictions that decrease appetite.

On average, patients with HF take seven prescribed medications per day, excluding over-the-counter or complementary and alternative medications, and the prevalence of polypharmacy is increasing^(28–32)^. The prevalence of excessive polypharmacy in patients with HF is about 2⋅5 times higher compared with community-dwelling older individuals^(33–35)^. Cobretti *et al.* found that 72 % of older patients with HF were taking more than ten medications, and 40 % were taking sixteen or more medications^(36)^. Polypharmacy could interfere with food consumption due to medication side effects, such as nausea, decreased appetite, dry mouth, and metallic taste^(37–40)^. For example, cardiac and anti-inflammatory medications cause taste dysfunction and are frequently linked to loss of appetite and anorexia^(41,42)^. Additionally, patients with diminished taste acuity may increase their sugar and/or fat intake^(43)^. To avoid the impact of polypharmacy on food intake, clinicians could (1) review medication history and each medication patients are currently taking, including over-the-counter or complementary and alternative medications, to identify medications that change patients’ appetites and to prevent possible interactions, (2) prescribe the most appropriate medication that is least likely to change patients’ appetites or alter the sense of smell or taste and (3) eliminate unnecessary medications to reduce the impact of polypharmacy on food intake among patients with HF^(44)^.

HF symptoms might also contribute to lower diet quality. Intestinal oedema that occurs with fluid overload can contribute to loss of appetite and decreased food intake^(45–47)^. Andreae *et al.* found decreased appetite in about one-third of patients with HF; lower functional capacity was associated with lower appetite^(48)^. Persistent shortness of breath can interfere with patients’ energy to prepare food, and they may purchase prepared foods that are often higher in fat, salt and sugar components compared with food cooked at home^(37,49)^. Lennie *et al.* reported fatigue as well as shortness of breath as factors that affect food intake in patients with HF when compared with healthy elders^(49)^. To decrease the negative impact of HF symptoms on diet quality, clinicians could focus on early detection of HF, monitor patients’ functional status and HF symptoms, and optimise symptom control.

Depressive symptoms are a major concern in HF. Approximately 20–40 % of patients with HF have depression and about half of them have significant depression^(50)^. The impact of depressive symptoms can result in lower diet quality due to a decrease in appetite or change in patients’ food preferences^(51–53)^. Fernstrom *et al.* reported an increased desire for carbohydrate and fat-rich foods in patients who were having depressive episodes compared with those who were feeling well^(52)^. Andreae *et al.* found a significant association between higher levels of depressive symptoms and poor appetite in patients with HF^(48)^. Thus, clinicians should periodically screen depressive symptoms, monitor their effect on change in food preferences and appetite, and intervene to reduce depressive symptoms in patients with HF.

Dietary restrictions might be another factor that lowers diet quality in patients with HF. Lennie *et al.* found that sodium and other diet restrictions affected food intake in patients with HF^(49)^. Although healthy elders also perceived sodium restriction to be a substantial barrier that affected food intake^(54)^, the impact of dietary restriction on food intake was greater in patients with HF than healthy elders^(49)^. Comorbidities, such as diabetes, chronic lung disease, ischaemic heart disease and chronic kidney disease, are common in patients with HF and require additional dietary restrictions, such as carbohydrates, proteins, fats, sodium and fluids, which can affect food intake^(55–57)^. Clinicians should consider referring their patients with multiple dietary restrictions to dieticians to help optimise their diet quality.

Our results in combination with prior research suggest that patients with HF are at risk for decreased food intake that can result in dietary deficiencies. If possible, healthcare providers should recommend patients to increase food intake or if not then encourage them to eat a more nutrient-dense diet, such as vegetables, whole grains and lean protein, and foods prepared with little or no saturated fats, added sugars and sodium^(58)^.

Our study has limitations. Our study was observational, which limits drawing cause and effect conclusions. However, we used a healthy older comparison group and a conditional logistic regression model which confirmed that HF status was associated with having low diet quality. Our study sample was mainly Caucasian. Thus, the results may not be generalisable to other populations with HF. There were differences between groups on variables that can influence food intake because these were not randomised samples. For example, people who eat often with somebody rather than alone consume more food. Thus, we included them in the conditional logistic regression model as covariates to control for their effects on food intake. We found group differences after controlling for these covariates, suggesting that the differences were independent of their influence. However, we could not include all potential confounders due to the limited sample size. Also, there might be unmeasured confounders. Because dietary record data were self-reported, there may be inaccuracies, including under-reporting^(59)^. To reduce inaccuracies, participants were given digital scales with detailed oral instruction by a research assistant, including paper instruction. Food models were also given to participants, so that they could estimate their serving sizes if they went out to eat where they could not use scales. Return demonstration of food measurement and recording food diary were also requested to ensure the accuracy of food diary recordings. Research nurses called participants to review the procedure and answer any questions they had on the first day of the food diary collection. Dietitians carefully reviewed food diaries with participants at the time of their visit to verify food preparation and serving sizes necessary to quantify nutrient analyses. In addition, we collected 4-day food diaries, including 1 weekend day and 3 weekdays, to account for day-to-day variations in food intake.

## Conclusions

Given that the patients with HF and healthy older adults were age-matched, our findings indicate that lower diet quality in patients with HF was a function of HF rather than age. HEI-2015 and nutrient density of diet were similar between patients with HF and healthy older adults. However, patients with HF had lower dietary intake compared with healthy older adults.

Importantly, we found that magnesium and vitamin C remained significantly lower in patients with HF compared with healthy older adults after we controlled for differences in food intake by referencing micronutrient intake per 1000 kcal. This suggests that patients with HF are at particular risk for deficiencies of these micronutrients. To avoid micronutrient deficiency, healthcare providers may need to encourage more nutrient-dense diets for patients with HF to compensate for decreased intake.
